# A reinforcement learning model for AI-based decision support in skin cancer

**DOI:** 10.1038/s41591-023-02475-5

**Published:** 2023-07-27

**Authors:** Catarina Barata, Veronica Rotemberg, Noel C. F. Codella, Philipp Tschandl, Christoph Rinner, Bengu Nisa Akay, Zoe Apalla, Giuseppe Argenziano, Allan Halpern, Aimilios Lallas, Caterina Longo, Josep Malvehy, Susana Puig, Cliff Rosendahl, H. Peter Soyer, Iris Zalaudek, Harald Kittler

**Affiliations:** 1 Institute for Systems and Robotics, LARSyS, Instituto Superior Técnico, Lisbon, Portugal; 2 Dermatology Service, Memorial Sloan Kettering Cancer Center, New York, NY USA; 3 Microsoft, Redmond, WA USA; 4 Department of Dermatology, Medical University of Vienna, Vienna, Austria; 5 Center for Medical Statistics, Informatics and Intelligent Systems (CeMSIIS), Medical University of Vienna, Vienna, Austria; 6 Ankara University School of Medicine, Department of Dermatology, Ankara, Turkey; 7 Second Department of Dermatology, Aristotle University of Thessaloniki, Thessaloniki, Greece; 8 Dermatology Unit, University of Campania, Naples, Italy; 9 Dermatology Unit, University of Modena and Reggio Emilia, Modena, Italy; 10 Azienda Unità Sanitaria Locale – IRCCS di Reggio Emilia, Centro Oncologico ad Alta Tecnologia Diagnostica-Dermatologia, Reggio Emilia, Italy; 11 Melanoma Unit, Dermatology Department, Hospital Clínic Barcelona, Universitat de Barcelona, IDIBAPS, Barcelona, Spain; 12 Centro de Investigación Biomédica en Red de Enfermedades Raras (CIBER ER), Instituto de Salud Carlos III, Barcelona, Spain; 13 General Practice Clinical Unit, Medical School, The University of Queensland, Brisbane, Queensland Australia; 14 Frazer Institute, The University of Queensland, Dermatology Research Centre, Brisbane, Queensland Australia; 15 Department of Dermatology, Medical University of Trieste, Trieste, Italy

**Keywords:** Cancer imaging, Physical examination, Skin manifestations

## Abstract

We investigated whether human preferences hold the potential to improve diagnostic artificial intelligence (AI)-based decision support using skin cancer diagnosis as a use case. We utilized nonuniform rewards and penalties based on expert-generated tables, balancing the benefits and harms of various diagnostic errors, which were applied using reinforcement learning. Compared with supervised learning, the reinforcement learning model improved the sensitivity for melanoma from 61.4% to 79.5% (95% confidence interval (CI): 73.5–85.6%) and for basal cell carcinoma from 79.4% to 87.1% (95% CI: 80.3–93.9%). AI overconfidence was also reduced while simultaneously maintaining accuracy. Reinforcement learning increased the rate of correct diagnoses made by dermatologists by 12.0% (95% CI: 8.8–15.1%) and improved the rate of optimal management decisions from 57.4% to 65.3% (95% CI: 61.7–68.9%). We further demonstrated that the reward-adjusted reinforcement learning model and a threshold-based model outperformed naïve supervised learning in various clinical scenarios. Our findings suggest the potential for incorporating human preferences into image-based diagnostic algorithms.

## Abstract (web summary)

A reinforcement learning model developed to adapt artificial intelligence (AI) predictions to human preferences showed better sensitivity for skin cancer diagnoses and improved management decisions compared to a supervised learning model.

## Main

Compared to clinical experts, artificial intelligence (AI)-based diagnostic methods have demonstrated similar or better accuracy in various areas of diagnostic imaging. As a result, AI-based decision-support tools are expected to facilitate access to expert-level image-based diagnostic accuracy^1–6^. To ensure the safety and effectiveness of AI-enabled medical devices, certain performance quality standards must be met. For example, regulations governing cancer diagnosis emphasize high sensitivity due to the greater potential harm of overlooking a malignancy compared to misclassifying a benign lesion as malignant. However, evaluating a diagnostic test based solely on sensitivity is inadequate as low specificity also poses risks, such as invasive procedures, patient anxiety and waste of healthcare resources. The trade-off between these harms differs depending on the type of cancer and is further influenced by human preferences, which refers to the personal judgments of physicians and patients regarding the relative value of potential outcomes within a specific clinical scenario. These preferences are not usually taken into account in AI training, but at best are implemented at more application-level logic through thresholds and cost-sensitive learning^7–9^.

Diagnostic procedures can be viewed as a sequential decision-making task in which a management decision is based on the likelihood of a potentially harmful diagnosis like cancer. In the field of diagnostic imaging, we can think of this as a Markov decision process where the initial states are image attributes, the possible actions are management strategies and the rewards are determined by the relative benefits and harms of diagnostic errors and appropriate and inappropriate management decisions. In this way, we can use reinforcement learning to find a strategy that maximizes cumulative rewards while considering clinician and patient preferences^10,11^.

To test whether reinforcement learning could be useful to adapt AI predictions to human preferences, we used the example of skin cancer diagnosis. This domain is challenging for AI because it involves imbalanced datasets dominated by benign conditions and represents a multiclass problem involving more than one type of cancer with different trade-offs^12^. Although less common than other skin cancers, melanoma has the highest mortality rate, and overlooking melanoma should carry a higher penalty than overlooking other types of skin cancer^13^.

First, we trained a supervised learning model (SL model) using a publicly available training set composed of 10,015 images including two types of skin cancer, melanoma and basal cell carcinoma, a precancerous condition (actinic keratosis/intraepidermal carcinoma) and four common benign conditions (nevi, benign keratinocytic lesions, dermatofibroma and vascular lesions)^14^. The model was trained to minimize a class-frequency weighted cross-entropy loss, with the goal to maximize average recall. The output of the model predicted multiclass probabilities for each of the seven diagnoses. The external validity of this model was tested on an independent test set of 1,511 images, where the model achieved an average accuracy of 77.8% with a sensitivity of 61.4% for melanoma (95% CI: 54.1–68.7%) and 79.6% for basal cell carcinoma (95% CI: 71.4–87.8%). This result is comparable to the results of above-average models obtained in an international competition using the same benchmark test set, and better than the results obtained by experts^3^. Although the model has acceptable multiclass accuracy, the low sensitivity for melanoma limits its use in clinical practice.

**Fig. 1:**
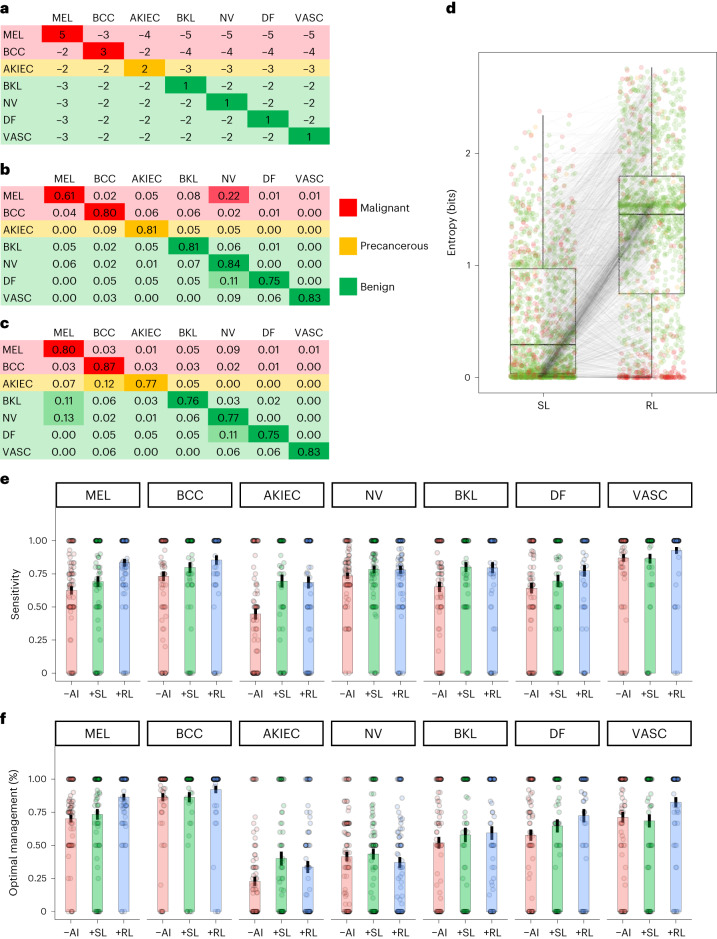
Comparison of models and reader study results. **a**, Expert-generated reward table used to train the RL model; rows, ground truth; columns, predictions. **b**,**c**, Confusion matrix of the SL model (**b**) and the RL model (**c**) using the same test set (*n* = 1511). Rows, ground truth; columns, predictions. The proportions are normalized by the row-sums (MEL: *n* = 171; BCC: *n* = 93; AKIEC: *n* = 43; BKL: *n* = 217; NV: *n* = 908; DF: *n* = 44; VASC: *n* = 35). **d**, Boxplot of difference in entropy of paired test set predictions (*n* = 1,511) of the SL model and the RL model. Black line, median; boxes, 25th–75th percentiles; whiskers, minimum and maximum values, *P* < 0.0001 (Wilcoxon test). **e**,**f**, Results of the reader study comparing sensitivities (**e**) and frequencies of optimal management decisions (**f**) of 89 dermatologists by diagnosis without AI support (−AI), with support by the SL model (+SL) and with support by the RL model (+RL). Optimal managements: ‘excision’ for melanomas and basal cell carcinomas; ‘local therapy’ for actinic keratoses/intraepidermal carcinoma; and ‘dismiss’ for nevi, benign keratinocytic lesions, dermatofibroma and vascular lesions. Bars, means; whiskers, standard error. Sample sizes: MEL(−AI): *n* = 89; MEL(+SL): *n* = 78; MEL(+RL): *n* = 81; BCC(−AI): *n* = 89; BCC(+SL): *n* = 63; BCC(+RL): *n* = 68; AKIEC(−AI): *n* = 89; AKIEC(+SL): *n* = 60; AKIEC(+RL): *n* = 72; NV(−AI): n = 89; NV(+SL): *n* = 88; NV(+RL): *n* = 85; BKL(−AI): *n* = 89; BKL(+SL): *n* = 65; BKL(+RL): *n* = 76; DF(−AI): *n* = 89; DF(+SL): *n* = 71; DF(+RL): *n* = 61; VASC(−AI): *n* = 89, VASC(+SL): *n* = 67; VASC(+RL): *n* = 65. Abbreviations: MEL, melanoma; BCC, basal cell carcinoma; AKIEC, actinic keratosis/intraepidermal carcinoma; BKL, benign keratinocytic lesion; NV, melanocytic nevus; DF, dermatofibroma; VASC, vascular lesion.

**Extended Data Fig. 1:**
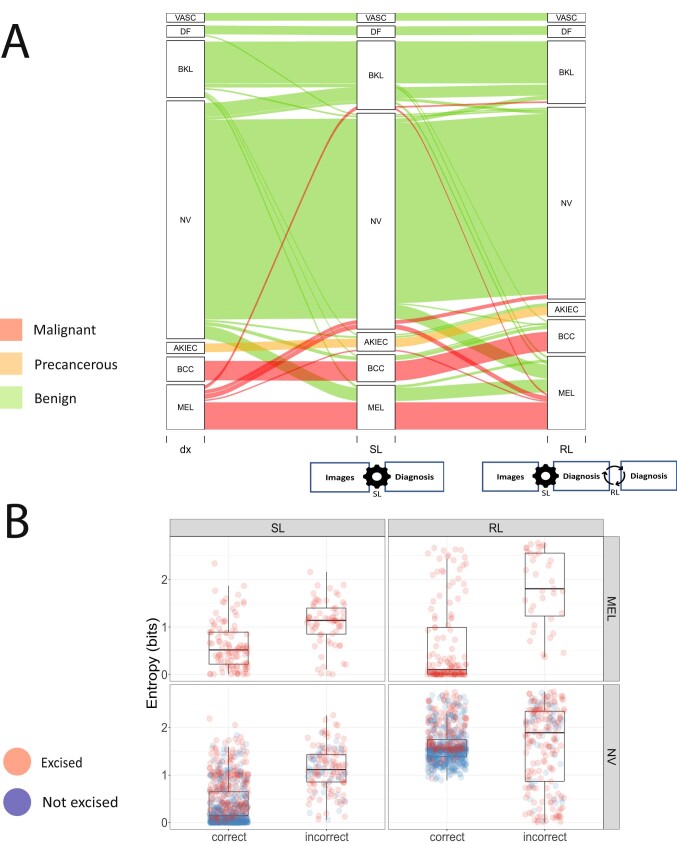
Comparison of baseline SL model with RL model. **a**: Alluvial plot of test set (n = 1511); the left block shows the ground truth, the middle block shows the results of supervised learning (SL), and the right block shows the results of reinforcement learning (RL) based on a reward table created by experts; Only alluvials with n > 5 are shown. MEL= melanoma (n = 171), BCC= basal cell carcinoma (n = 93), AKIEC= actinic keratosis and intraepidermal carcinoma(n = 43), BKL= benign keratinocytic lesion (n = 217), NV= melanocytic nevus (n = 908), DF=dermatofibroma (n = 44), VASC= vascular lesion (n = 35). **b**: Boxplots of entropy of correct and incorrect predictions for melanoma (n = 171) and melanocytic nevi (n = 908) according to applied model. Black line = median, boxes = 25th–75th percentiles, whiskers = values within 1.5 times interquartile range; Abbreviations: SL= supervised learning, RL= reinforcement learning, dx =ground truth.

Next, we set up a reinforcement learning model (RL model) with deep *Q*-learning using a one-dimensional vector combining the multiclass probabilities and the feature vector of the SL model as the initial state^11^. We used a dermatologist-generated reward table in which rewards and penalties for correct and incorrect diagnoses depend on the type of skin cancer (Fig. 1a). Using the same training and test sets, the RL model achieved a significantly higher sensitivity for melanoma (79.5%, 95% CI: 73.5–85.6%, *P* < 0.001) and for basal cell carcinoma (87.1%, 95% CI: 80.3–93.9%, *P* < 0.001) compared to the baseline SL model while maintaining a high average accuracy of 79.2% (Fig. 1b,c). This increase in sensitivity for melanoma was mainly driven by reclassifying melanomas diagnosed as nevi by the SL model (Extended Data Fig. 1a).

We also calculated the Shannon entropy of AI predictions and used it as a marker of model uncertainty. We found that the RL model increased the entropy of predictions in comparison to the SL model (median: 0.30 bits, 25th–75th percentile: 0.04–0.97 bits versus median: 1.46 bits, 25th–75th percentile: 0.75–1.80 bits, *P* < 0.001; Fig. 1d). While this increase in uncertainty has no decremental effect on average accuracy, it reduces the overconfidence of AI predictions when the diagnosis is incorrect (median: 1.13 bits, 25th–75th percentile: 0.82–1.49 bits for 298 cases incorrectly classified by the SL model versus 1.81 bits, 25th–75th percentile: 0.90–2.32 bits for 333 cases incorrectly classified by the RL model, *P* < 0.001). While the addition of human preferences increased the uncertainty of predictions on average, it decreased the uncertainty for melanomas if they were correctly predicted by the RL model (Fig. 1d and Extended Data Fig. 1b).

Next, we investigated the utility of the RL model for management decisions in a human-in-the-loop scenario. We conducted a reader study with 89 dermatologists who had to diagnose the same image with and without AI support and determine management, choosing between four treatment decisions: dismiss, excise, treat locally or monitor. For AI support, dermatologists were alternately offered the multiclass probabilities of the SL or the RL model. The rate of correct diagnoses increased from 68.0% (95% CI: 65.3–70.6%) without AI support to 75.3% with SL model support (mean difference +7.3%, 95% CI: 4.6–10.2%, *P* < 0.001) and to 79.9% with RL model support (mean difference +12.0%, 95% CI: 8.8–15.1%, *P* < 0.001). The readers’ sensitivity for melanoma improved from 62.4% (95% CI: 56.3–68.6.0%) without support to 69.4% (95% CI: 61.3–77.0%, *P* < 0.001) with SL model support and to 83.9% (95% CI: 77.7–89.0%, *P* < 0.001) with RL model support. The sensitivity for basal cell carcinoma was similarly improved while the sensitivity for other diagnoses did not decrease substantially (Fig. 1e). Furthermore, management decisions of expert readers improved with AI support (Fig. 1f). The proportions of optimal management decisions increased from 57.4% (95% CI: 54.2–60.5%) without AI support to 61.7 (95% CI: 58.0–65.3%, *P* = 0.03) with SL model support and to 65.3% (95% CI: 61.7–68.9%, *P* < 0.001) with RL model support. This improvement was most pronounced for melanoma (without AI: mean = 70.1%, 95% CI: 64.5–75.7%; SL model support: mean = 73.4%, 95% CI: 65.5–81.2%, *P* = 0.51, and RL model support: mean = 86.4%, 95% CI: 81.5–91.4%, *P* < 0.001).

**Fig. 2:**
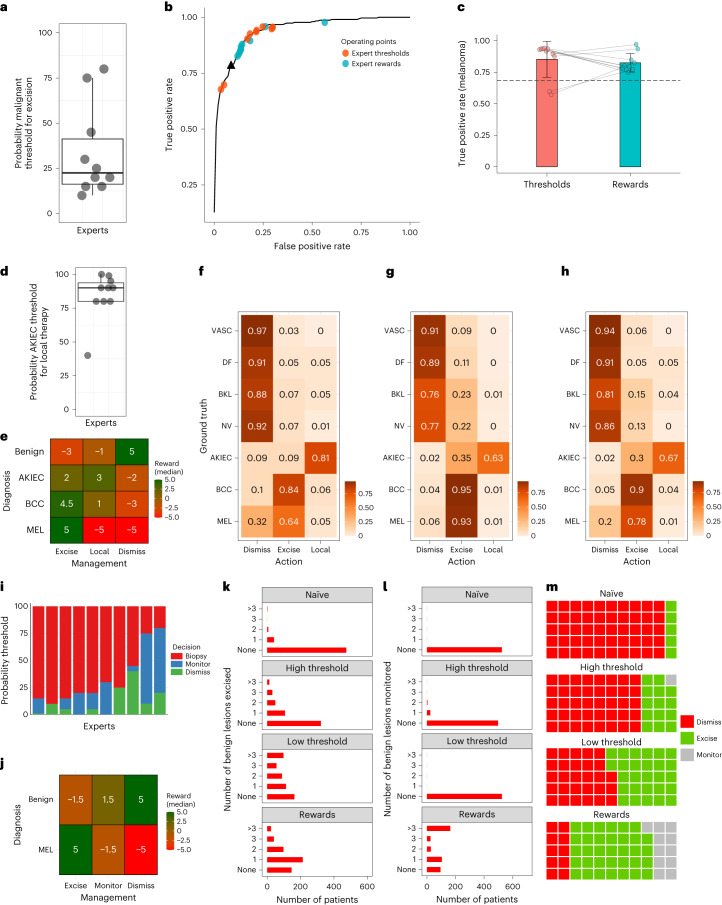
Comparison of models in three different scenarios. Top level (binary scenario: benign versus malignant): **a**, Experts’ malignancy probability thresholds for decision to excise (*n* = 10). Lines, median; boxes, 25th–75th percentiles; whiskers, values within 1.5 times interquartile range. **b**, Receiver operating characteristic curve derived from the SL model and operating points of ten experts using either thresholds (SL model) or rewards (RL model). Possible management decisions were ‘dismiss’ or ‘excise’. True and false positive rates refer to proportions of malignant and benign lesions that were excised. Black triangle, naïve approach (excision if malignant probability > 0.5). **c**, Boxplot comparing TPRs for melanomas applying thresholds (SL model) and rewards (RL model) provided by ten experts. Bars, means; whiskers, standard deviations (*P* = 0.11, paired *t*-test); dashed line, naïve approach. Middle level (multiclass scenario, additional therapeutic option): **d**, Thresholds of ten experts for probabilities of actinic keratosis/intraepidermal carcinoma for decision to treat locally. Line, median; boxes, 25th–75th percentiles; whiskers, values within 1.5 times interquartile range. **e**, Median rewards per action and diagnosis. **f**–**h**, Confusion matrices of actions by diagnosis: naïve approach (**f**), threshold-adjusted SL model (**g**) and RL model (**h**). Lower level (patient-centered approach, 7,375 lesions, 524 patients): **i**, Thresholds of ten experts for malignancy probabilities for decision to dismiss, monitor or excise. **j**, Median rewards per action and diagnosis. **k**, Number of excisions of benign lesions by patient according to model. **l**, Number of monitored benign lesions by patient according to model. **m**, Management strategies for 55 melanomas according to model.

Finally, we compared the reward-based RL model with a threshold-based SL model and a naïve model that simply chooses the optimal management strategy according to the top 1 class prediction of the SL model. To this end, we created three different clinical scenarios and used thresholds and rewards provided by ten experts in the field of skin cancer diagnosis (Fig. 2).

For the simplest scenario, we divided the data into a malignant (melanoma, basal cell carcinoma, actinic keratosis/intraepidermal carcinoma) and a benign class (nevi, vascular lesions, dermatofibroma and benign keratinocytic lesions) and considered only two treatment options, either ‘dismiss’ or ‘excision’. In this scenario, the proportion of malignant lesions that were managed by excision represented the true positive rate (TPR). As shown in Fig. 2b, the threshold-adjusted SL model and the reward-based RL model caused a shift in operating points on the receiver operating curve, bringing them closer to regions with an increased TPR. While the TPR was 78.2% for the naïve approach, it increased to 88.9% (95% CI: 80.9–96.9%) for the threshold-adjusted SL model and to 88.0% (95% CI: 83.4–92.5%) for the RL model. As shown in Fig. 2c, the TPR for melanoma was 68.4% for the naïve approach, 85.4% (95% CI: 74.7–96.0%) for the threshold-adjusted SL model and 82.5% (95% CI: 75.7–89.3%) for the RL model. The difference between the two models was not significant (*P* = 0.11).

**Extended Data Fig. 2:**
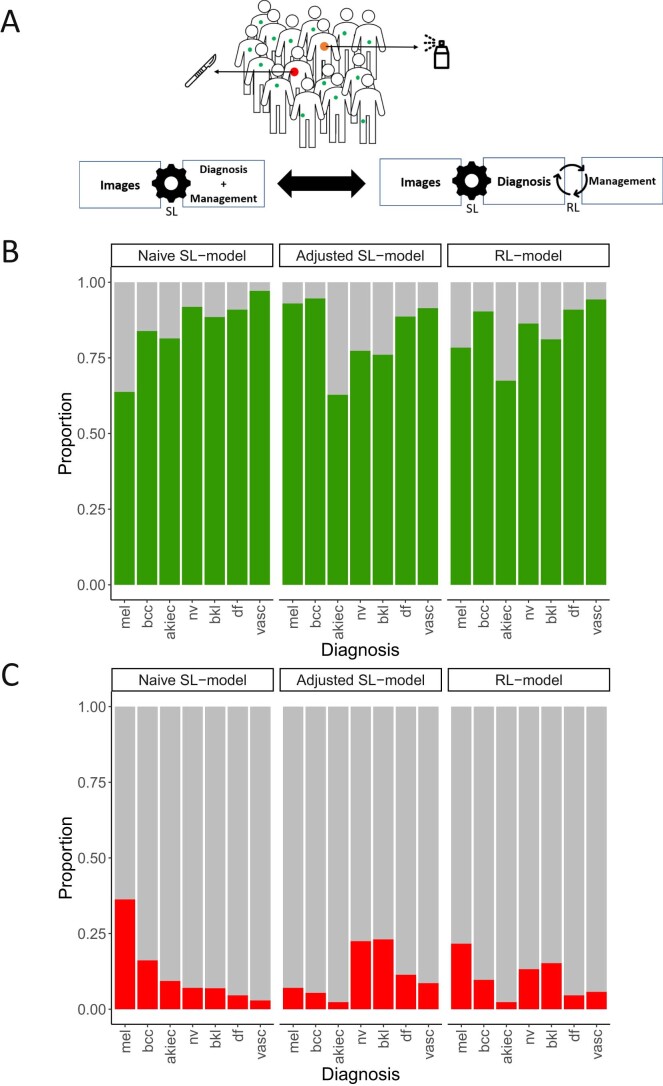
Scenario with 7 diagnoses and ‘local therapy’ as an additional treatment option. **a**: Graphical abstract of scenario adding the treatment option ‘local therapy’ (for example cryotherapy) for actinic keratosis/intraepidermal carcinomas. While excision is the optimal management for melanoma and most basal cell carcinomas, local therapy is optimal for actinic keratosis/intraepidermal carcinoma. We judged local therapy to be a harmful treatment for melanomas and suboptimal for basal cell carcinomas suitable for surgery (all basal cell carcinomas in the dataset). **b**: Proportion of cases per diagnosis and model that received optimal management (excision for melanoma and basal carcinoma, local therapy for actinic keratoses/intraepidermal carcinoma, and no treatment (‘dismiss’) for all benign diagnoses); **c**: Proportion of cases per diagnosis and model that were mismanaged. Mismanagement included all procedures except excision for melanoma and basal cell carcinoma, all procedures except excision or local therapy for actinic keratoses/intraepidermal carcinoma, and all procedures except ‘dismiss’ for all benign conditions (nevus, benign keratinocytic lesions, dermatofibroma, and vascular lesions). Abbreviations and sample size: mel= melanoma (n = 171), bcc= basal cell carcinoma (n = 93), akiec= actinic keratosis/intraepidermal carcinoma(n = 43), bkl= benign keratinocytic lesion (n = 217), nv= nevus (n = 908), df=dermatofibroma (n = 44), vasc= vascular lesion (n = 35).

In a second scenario, we explored all seven diagnoses and added local therapy as a treatment option. While excision is the optimal management for melanoma and most basal cell carcinomas, local therapy is optimal for actinic keratosis/intraepidermal carcinoma. For this scenario, we used the median values of the expert estimates for both rewards and thresholds. We found that the threshold- and reward-based model were superior to the naïve model in increasing the frequency of the optimal management decision as well as in preventing mismanagement of malignant lesions (Fig. 2c–e and Extended Data Fig. 2). In the 307 malignant conditions that require treatment, mismanagement was at 21.8% in the naïve approach (95% CI: 17.2–26.4%), 13.4% in the RL model (95% CI: 9.8–17.7%) and 5.2% in the threshold-adjusted SL model (95% CI: 3.0–8.3%, *P* < 0.0001).

**Extended Data Fig. 3:**
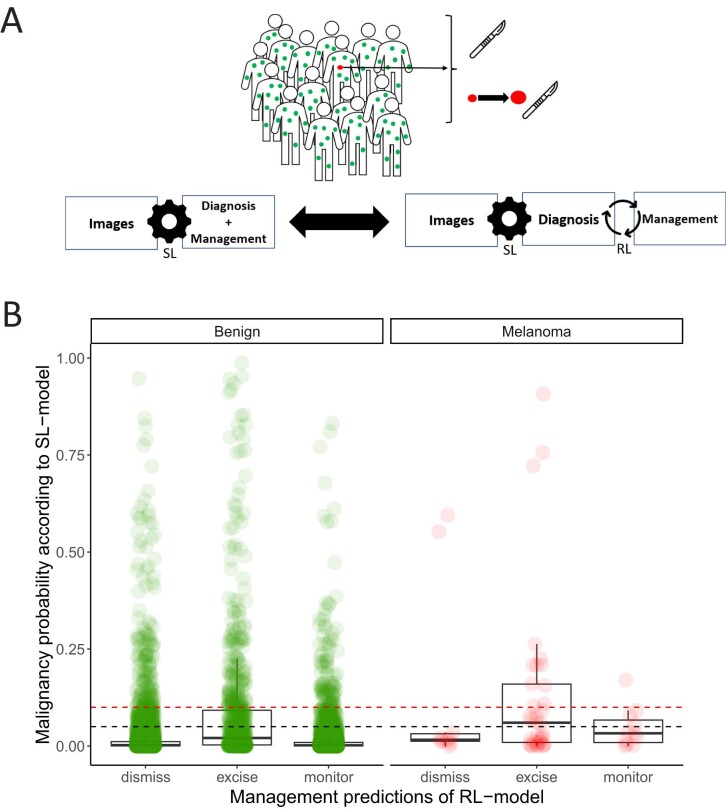
Scenario of high-risk patients with multiple nevi. **a**: Graphical abstract of scenario of monitoring of high-risk individuals with multiple nevi. Due to the large number of lesions per patient, this scenario requires a more patient-centered and less lesion-centered approach. Most melanomas detected during monitoring are noninvasive, slow-growing lesions. Short-term monitoring of these melanomas, while not optimal, is considered acceptable. **b**: Malignancy probability predictions of the baseline SL model according to management predictions of the RL model for benign lesions (n = 7320) and melanomas (n = 55). The red dashed horizontal line indicates the median value of the melanoma probability selected by 10 experts as threshold for excision. The black dashed horizontal line indicates the minimum value. Black line = median, boxes = 25th–75th percentiles, whiskers = values within 1.5 times the interquartile range.

The most complex scenario involved monitoring of high-risk individuals with multiple nevi. Nevi are not only indicators of melanoma risk but also are potential precursors or may have morphologic criteria similar to melanoma. Most melanomas detected during monitoring are noninvasive, slow-growing lesions that mimic nevi. Short-term monitoring of these melanomas, while not optimal, is considered acceptable, as reflected in the moderate penalty set by the experts for this procedure (Fig. 2j). Because this scenario requires a more patient-centered and less lesion-centered decision-making approach, we created an RL model in which each episode consisted of all lesion images of a single patient to maximize the cumulative reward per patient. Here, as before, we used the median values of the expert estimates, except for the low-threshold model, for which we used the minimum value. In a test set of 7,375 lesions (7,320 benign lesions (98.5% nevi) and 55 noninvasive or microinvasive melanomas) from 524 patients (median: 12 lesions per patient, range: 6–51), the naïve approach would remove 9.1% (*n* = 5) of melanomas, while two patients (0.4%) would have >3 benign lesions removed. The threshold approach would remove 25.5% (*n* = 14) of melanomas and >3 benign lesions in 13 patients (2.4%). As shown in Fig. 2k, lowering the threshold results in a high number of patients with >3 excised benign lesions (*n* = 98, 18.6%) and with an increase of excised melanomas (49.1%, *n* = 27). The RL model would remove 61.8% (*n* = 34) and monitor 20% (*n* = 11) of melanomas, outperforming all other models in terms of acceptable management decisions for these melanomas (Extended Data Fig. 3). At the same time, 23 patients (4.4%) would have >3 benign lesions removed. A distinctive feature of the RL model would be the high number of benign lesions (41.6%, *n* = 3,045) that are monitored (Fig. 2f–h). This strategy aligns with the practices of expert clinicians when monitoring high-risk patients, aiming at reducing the number of missed melanomas while keeping the number of excisions within an acceptable range.

Here, we demonstrate that the integration of human preferences, represented as reward tables created by experts, enhances the performance of a pretrained AI decision-support system. Improvement is evident in both the system’s standalone performance and its ability to collaborate effectively with dermatologists. Dermatologists’ improvement may be due to the RL model reducing AI overconfidence by considering consequences of management decisions. We further show that incorporating human preferences improves management decisions in complex clinical scenarios. This optimization of medical decision-making has traditionally been captured by risk–benefit analysis, but due to the complexity of this method, individualized medical decision-making is not yet attainable^15^. The current trend toward AI-based decision support in medicine presents an opportunity to implement individualized medical decision-making in clinical practice. However, this can only happen if the concept of incorporating human preferences is also given greater consideration in the development of such systems.

Based on our results, we suggest that RL, among other techniques, could be a suitable tool for this purpose, although it is not necessarily the best solution. A limitation of the RL method is that the model must be retrained, whereas other simpler approaches, such as thresholding, can be applied without retraining. As demonstrated in our binary scenario, both methods—that is, the threshold method and the RL method—will improve management decisions compared to the naïve SL model by optimizing operating points on a decision curve. Another limitation is that we included only physicians’ but not patients’ preferences. There is growing emphasis on patient-centered care, where the preferences and needs of patients are considered. For future clinical applications, we envision physicians and patients collaborating in shared medical decision-making to jointly develop reward tables. Creating reward tables would provide a secondary benefit of making rewards explicit and transparent, enhancing the acceptance of AI decision-support tools. Our study focused on management decisions related to skin cancer diagnosis. Although the basic concepts can be applied to other diagnostic scenarios, those outside diagnostic medicine may require different approaches.

In conclusion, our study shows that incorporating human preferences can improve AI-based diagnostic decision support and that such preferences could be considered when developing AI tools for clinical practice. RL could be a potential alternative to threshold-based methods for creating tailored approaches in complex clinical scenarios. However, additional research, including evaluating patient and provider satisfaction, is necessary to fully uncover the potential of RL in this context.

## Methods

### Supervised learning and reinforcement learning

For the supervised learning, we fine-tuned a convolutional neural network for classification of seven different categories of the HAM10000 dataset, as described previously^14^. For RL, we created a deep *Q*-learning model consisting of a multilayer perceptron that receives as input a one-dimensional state vector from the feature vectors and probabilities of the supervised model. For the patient-centered scenario, we normalized the input vector to account for the context of multiple lesions (the lesion state vector was divided position-wise by the average across all lesion vectors of the same patient). Python language (v.3.8) was used to conduct all experiments. The RL models were implemented using TensorFlow v.2.8, together with a set of packages: NumPy (v.1.20.3), Scikit-Learn (v.1.1.2), pandas (v.1.3.4) and OpenAI Gym (v.0.23.1).

The RL models predict the *Q*-value for each possible action. Depending on the RL model, the action space was either selecting a diagnosis (seven actions) or selecting a management option, ranging from two actions (dismiss or excise) to four actions (dismiss, monitor, treat locally and excise), depending on the type of scenario. The RL models were trained following Mnih et al.^11^ using an exploration–exploitation strategy, a replay buffer and a target *Q*-network with a lower update rate to stabilize the training process^11^. Huber loss was adopted as the loss function and the weights of the *Q*-network were updated using the Adam optimizer with a learning rate of 0.025. To improve generalization, we added dropout layers with a probability of 0.05. We tested different configurations for the *Q*-network (number and size of the hidden layers and combination of the input state), buffer size, episode length, update rates for the *Q*-network and the target model, and exploration *ε*. The best *Q*-network models consisted of a multilayer perceptron with a 256-unit fully connected layer with a ReLu activation that processes the features of the supervised model, followed by the concatenation of its output with the logits. The concatenation is fed to the output layer, which has the same units as the number of possible actions and a linear activation. The replay buffer size was set to 10,000 and the update rates for the *Q*- and target networks were set to 4 and 8,000 iterations, except in the patient-centered model where the updates were set to 35 and 5,800 iterations. We also ran experiments with several episode lengths, ranging from 250 to 12, except in the patient-centered model where the episodes had a varying length depending on the number of lesions per patient. We found that the episode length had a marginal effect on the performance of the RL model. In the case of the patient-centered model, we found that ordering the lesions according to malignancy probability inside the episode led to better performances. Finally, the *ε* was set to 0.2. We also found that modifications to the reward table resulted in only minor changes or degradation of results compared to the originally designed reward table.

We used the HAM10000 dataset to train all RL models, except in the patient-centered scenario^1^. To track the evolution of the models, we split the original HAM10000 set into a single 80/20 partition, of which the latter was used as the validation set. Because of the relatively small number of patients and the high variability in the number of lesions per patient, the patient-centered dataset was used to train and evaluate the RL model based on a 20-fold cross-validation strategy.

The reward table for the basic RL model was created in advance in consensus by three expert dermatologists (H.K., P.T., V.R.). To compare the reward model with the threshold model in different clinical scenarios, we asked 12 dermatologists with extensive experience in treating neoplastic skin lesions to provide us with their reward tables and thresholds for each scenario. Because two of the 12 experts provided incomplete information (they did not specify thresholds for either the binary scenario or the scenario with the additional treatment option), we had a total of ten expert assessments available. Treatment decisions using the threshold model followed a preference-based hierarchy. The model initially determined if the predicted melanoma probability exceeded the excision threshold. If not, it considered the overall malignancy probability and then the probabilities of basal cell carcinoma and actinic keratosis/intraepidermal carcinoma. The median values of the rewards and thresholds were used for the SL model and the RL model, respectively. For the low-threshold approach in the patient-centered scenario, we used the minimum value rather than the median value of the ten thresholds reported by the experts.

### Entropy

We calculated the Shannon entropy as a measure of uncertainty in the predictions of the machine learning models using the following formula, where H is entropy, X is a discrete random variable with possible probabilities (p) ranging from p_1_ to p_n_, and i is an index variable:

$$H\left(X\right)=H\left({p}_{1},\ldots .,{p}_{n}\right)=-\mathop{\sum }\limits_{i=1}^{n}{p}_{i}\log 2{p}_{i}$$

### Datasets

The publicly available HAM10000 dataset was used to train the SL model and the RL model^1^.

The ISIC 2018 challenge test set was used as an independent test set for the reader study and for the external validation of the SL model and the RL model^3^.This set includes 1,511 retrospectively collected dermatoscopic images from different sites including Austria (*n* = 928), Australia (*n* = 267), Turkey (*n* = 117), New Zealand (*n* = 87), Sweden (*n* = 92) and Argentina (*n* = 20) to ensure diversity of skin types. The mean age of patients was 50.8 years (s.d.: 17.4 years), and 46.2% of patients were female. The ground truth was routine pathology evaluation (*n* = 786), biology (that is, >1.5 years of sequential dermatoscopic imaging without changes; *n* = 458), expert consensus in inconspicuous cases that were not excised or biopsied (*n* = 260) and in vivo confocal images (*n* = 7). Fewer than ten cases with ambiguous histopathologic reports were excluded. For the patient-centered scenario, we used dermatoscopic images of 7,375 lesions from 524 patients (mean: 51.1 years, s.d.: 11.8 years, 46.6% females). Images were collected either at the University Department of Dermatology, Medical University of Vienna (*n* = 4,839) or at a dermatology practice in Vienna (*n* = 2,536). The consecutive dataset included 55 melanomas, all of which were either noninvasive (in situ) or microinvasive (<0.8 mm invasion thickness, tumor stage T1a). Most benign lesions that were selected for monitoring by the treating dermatologists were nevi (*n* = 7,213). The remaining benign lesions were keratinocytic lesions (*n* = 53), dermatofibromas (*n* = 31), or vascular lesions (*n* = 20) and other benign lesions (*n* = 3).

### Interaction platform, raters and reader study

We used the web-based platform DermaChallenge, which was developed at the Medical University of Vienna, as the interface for the reader study^16^.The platform is split into a back end and front end, and both are deployed on a stack of standard web technologies (Linux, Apache, MariaDB and PHP). The front end is optimized for mobile devices (mobile phones and tablets) but can also be used on any other platform via a JavaScript-enabled web browser. Readers were recruited by using mailing lists and social media posts from the International Society of Dermoscopy. To participate in the study, raters had to register with a username, a valid email address and a password. In addition, we asked for age (age groups spanning 10 years), sex, country and profession. The readers’ task was to diagnose the unknown test images first without and then with decision support based on either the SL model or the RL model. The images were presented in batches of ten selected randomly from the test set of 1,511 images. We drew a stratified random sample to ensure a predefined class distribution of three nevi, two melanomas and one example of each other class. Readers could repeat the survey with different batches at their own discretion. The study was online from 17th November 17th 2022 to 2nd February 2023. During this time, we collected 613 complete tests from 89 dermatologists.

### Statistical analysis

Comparisons of continuous data between groups were performed with paired or unpaired *t*-tests or Wilcoxon signed-rank tests, as appropriate. Chi-square tests or McNemar tests were used for proportions. Reported *P* values are two-sided and a *P* value < 0.05 was regarded as statistically significant. All analyses were performed with R Statistics v.4.2.1 (ref. ^17^) and plots were created with ggplot2 v.3.3.6.

### Ethics statement and informed consent

This project was conducted after ethics review by the Ethics Review Board of the Medical University of Vienna (Protocol No. 1804/2017, Amendment 4th April 2022). When registering, all participants of the reader study platform agreed that their data could be used for scientific research and were made aware that they could revoke this consent at any time. Readers received no compensation for their participation.

### Reporting summary

Further information on research design is available in the Nature Portfolio Reporting Summary linked to this article.

## Online content

Any methods, additional references, Nature Portfolio reporting summaries, source data, extended data, supplementary information, acknowledgements, peer review information; details of author contributions and competing interests; and statements of data and code availability are available at 10.1038/s41591-023-02475-5.

## Supplementary information

Reporting Summary

## Data Availability

The origin of the training set images is reported in the dataset-publication of HAM10000 in Nature Scientific Data ^14^. Training set images are available from the ISIC Image Archive at https://api.isic-archive.com/collections/66/ or the Harvard Dataverse at 10.7910/DVN/DBW86T (ref. ^18^). Test set images are available from the ISIC Image Archive at https://challenge.isic-archive.com/data/#2018. The ISIC image archive initially featured a test set comprising 1,512 images, but for this research, one image known as the ‘easter egg’ (ISIC_0035068) was excluded. The ground truth of the test set images is available from the Harvard Dataverse at 10.7910/DVN/DBW86T (ref. ^18^). Anonymous reader data of the test set images and the entire image dataset used in the patient-centered model can be downloaded from the Harvard Dataverse at 10.7910/DVN/PWQMQ7 (ref. ^19^).

## Code availability

The code for the supervised learning model is available athttps://github.com/ptschandl/dermatoscopy_resnet34_nmed_2020. The code for the reinforcement learning model is available at https://github.com/catarina-barata/Skin_RL.
