# Supplementary material for: A reinforcement learning model for AI-based decision support in skin cancer
Source: Nat Med. 2023 Jul 27;29(8):1941–6. doi: 10.1038/s41591-023-02475-5 (PMC10427421; doi:10.1038/s41591-023-02475-5)
Supplement: Supplementary file 1 — Reporting Summary [file 41591_2023_2475_MOESM1_ESM.pdf]

nature portfolio

Corresponding author(s): Harald Kittler

Last updated by author(s): Jun 19, 2023

Reporting Summary

Nature Portfolio wishes to improve the reproducibility of the work that we publish. This form provides structure for consistency and transparency in reporting. For further information on Nature Portfolio policies, see our Editorial Policies and the Editorial Policy Checklist.

Statistics

For all statistical analyses, confirm that the following items are present in the figure legend, table legend, main text, or Methods section.

n/a	Confirmed	
☐	☒	The exact sample size (*n*) for each experimental group/condition, given as a discrete number and unit of measurement
☐	☒	A statement on whether measurements were taken from distinct samples or whether the same sample was measured repeatedly
☐	☒	The statistical test(s) used AND whether they are one- or two-sided<br>*Only common tests should be described solely by name; describe more complex techniques in the Methods section.*
☐	☒	A description of all covariates tested
☐	☒	A description of any assumptions or corrections, such as tests of normality and adjustment for multiple comparisons
☐	☒	A full description of the statistical parameters including central tendency (e.g. means) or other basic estimates (e.g. regression coefficient) AND variation (e.g. standard deviation) or associated estimates of uncertainty (e.g. confidence intervals)
☐	☒	For null hypothesis testing, the test statistic (e.g. F, t, r) with confidence intervals, effect sizes, degrees of freedom and P value noted<br>*Give P values as exact values whenever suitable.*
☒	☐	For Bayesian analysis, information on the choice of priors and Markov chain Monte Carlo settings
☒	☐	For hierarchical and complex designs, identification of the appropriate level for tests and full reporting of outcomes
☐	☒	Estimates of effect sizes (e.g. Cohen's *d*, Pearson's *r*), indicating how they were calculated

Our web collection on statistics for biologists contains articles on many of the points above.

Software and code

Policy information about availability of computer code

Data collection	Python language (version 3.8) was used to conduct all experiments. The RL models were implemented using Tensorflow 2.8, together with a set of packages: numpy (v1.20.3), scikit-learn (v1.1.2), pandas (v1.3.4), and open ai gym (v0.23.1). The supervised model was implement using PyTorch and had been previously used in publications, as reported in the "Methods" section of our submission. Data of the reader study was collected using a web-based platform using PHP (v7.1) and MariaDB (vS.5.60). The openSource PHP Framework Laravel (vS.5) and open Source JavaScript Framework ReactJS (v16.9) were used to implement the study platform. The code for supervised learning model is available at https://github.com/ptschandl/dermatoscopy_resnet34_nmed_2020. The code for the reinforcement learning model is available at https://github.com/catarina-barata/Skin_RL.
Data analysis	The results from the RL models were analyzed using standard scikit-learn (1.1.2) functions that allow the computation of evaluation metrics such as the confusion matrix, sensitivity scores, and balanced accuracy. A more detailed statistical analysis was performed using R Statistics R v4.2.1, using packages dplyr v1.0.7 for data manipulation, and epiR v2.0.39 for diagnostic values. Plots were created with ggplot2 v3.3.6.

For manuscripts utilizing custom algorithms or software that are central to the research but not yet described in published literature, software must be made available to editors and reviewers. We strongly encourage code deposition in a community repository (e.g. GitHub). See the Nature Portfolio guidelines for submitting code & software for further information.

Data

Policy information about availability of data

All manuscripts must include a data availability statement. This statement should provide the following information, where applicable:

- Accession codes, unique identifiers, or web links for publicly available datasets
- A description of any restrictions on data availability
- For clinical datasets or third party data, please ensure that the statement adheres to our policy

Origin of training set images is reported in the dataset-publication of HAM10000 in Nature Scientific Data (Tschandl, P., Rosendahl, C. & Kittler, H. The HAM10000 dataset, a large collection of multi-source dermatoscopic images of common pigmented skin lesions. Sci Data 5, 180161, 2018). Training set images are available from the ISIC Image Archive at https://api.isic-archive.com/collections/66/ or the Harvard Dataverse at https://doi.org/10.7910/DVN/DBW86T. Test set images are available from the ISIC Image Archive at https://challenge.isic-archive.com/data/#2018. The ISIC image archive initially featured a test set comprising 1512 images, but for this research, one image known as the "easter egg" (ISIC_0035068) was excluded. The ground truth of the test set images is available from the Harvard Dataverse at https://doi.org/10.7910/DVN/DBW86T. Anonymous reader data of the test set images and the entire image dataset used in the patient-centered model can be downloaded from the Harvard Dataverse at https://doi.org/10.7910/DVN/PWQMQ7.

Human research participants

Policy information about studies involving human research participants and Sex and Gender in Research.

Reporting on sex and gender	To participate in the study, raters had to register with a username, a valid e-mail address and a password. They were also asked for age (age groups of 10), biologic sex, country, and occupation. The options for sex were male, female, or other. We decided not to include the age, biologic sex, and country information in the reader description because these attributes are irrelevant to expertise.
Population characteristics	We recruited 101 dermatologists for the online reader study, of whom 89 completed at least one test round. Of the 89 readers, 52 were females. We recruited 58 dermatology residents, and 31 board certified dermatologists. Of the 89 readers, 47 were between 24-33 years old, 24 between 34-43 years, 9 between 44-53 years, 8 between 54-and 63 years, and one reader was older than 63 years. The expertise of each rater was measured by screening tests consisting of simple domain-specific tasks involving assigning cases to one of 7 possible diagnoses.
Recruitment	Mailings and social media posts of the International Dermoscopy Society were used to recruit targeted groups. The recruitment was focused on dermatologists. It is possible that recruitment of raters is influenced by self-selection bias and therefore biased towards the selection of motivated and skilled raters. Because of self selection bias, the generalisability of our results to a less motivated group of readers may be limited. When registering, all participants of the reader study platform agreed that their usage data may be used for scientific research and they can revoke this consent at any time. Readers received no compensation for their participation.
Ethics oversight	Ethics review board of the Medical University of Vienna, Protocol No. 1804/2017, Amendment April 4th, 2022

Note that full information on the approval of the study protocol must also be provided in the manuscript.

Field-specific reporting

Please select the one below that is the best fit for your research. If you are not sure, read the appropriate sections before making your selection.

☐ Life sciences ☒ Behavioural & social sciences ☐ Ecological, evolutionary & environmental sciences

For a reference copy of the document with all sections, see nature.com/documents/nr-reporting-summary-flat.pdf

Behavioural & social sciences study design

All studies must disclose on these points even when the disclosure is negative.

Study description	Quantitative experimental research data. In the online reader study the readers' tasks were to diagnose the unknown test images and to suggest a management strategy first without and then with decision support based on either the SL-model or the RL-model. The images were presented in batches of 10 images selected randomly from the test set of 1511 images.
Research sample	Participants of the reader study are users of a public open reader platform. They were recruited using mailing lists and social media posts from the International Society of Dermoscopy. The recruitment was focused on dermatologists and dermatology residents. It is possible that recruitment of raters is influenced by self-selection bias and therefore biased towards the selection of motivated and skilled raters. Because of self selection bias, the generalisability of our results to a less motivated group of readers may be limited. We recruited 101 readers for the online reader study, of whom 89 completed at least one test round. To participate in the study, raters had to register with a username, a valid email address, and a password. In addition, we asked for age (age groups spanning 10 years), sex, country and profession. Of the 89 readers, 52 were females. We recruited 58 dermatology residents, and 31 board

	certified dermatologists. Of the 89 readers, 47 were between 24-33 years old, 24 between 34-43 years, 9 between 44-53 years, 8 between 54-and 63 years, and one reader was older than 63 years. Origin of training and test set images is reported in the dataset-publication of HAM10000 in Nature Scientific Data (doi: 10.1038/sdata.2018.161) and The Lancet Oncology (doi: 10.1016/S1470-2045(19)30333-X).
Sampling strategy	Data collection for the reader study was performed prospectively. The sample of readers is a convenience sample by self-selection among suitable candidates (dermatologists). Based on findings in a previous study (doi.org/10.1038/s41591-020-0942-0) we observed an improvement of 13.5% with AI support with a standard deviation of 16,4%. Given this effect size and a power of 0.8 at a two-sided p-value <.05, more than 15 readers would need to be available for analysis (paired T-test). We finally recruited 89 raters.
Data collection	Data was recorded in a web-based training and study platform hosted by the Medical University of Vienna. The researchers were not present during interaction experiments, as raters could participate on any device (including smartphones, tablets, laptops and desktop computers) with internet connection and a JavaScript-enabled browser. The data collected from each reader included age, sex, country, and profession. Each rater had to perform multiple screening tests to ensure that the self-reported experience matched actual skills. During the experiments the readers' task was to diagnose the unknown test images first without and then with decision support based on either the SL-model or the RL-model. We collected the given diagnosis (single choice from 7 possible diagnoses), the selected management (single choice from 4 possible actions), and the time needed with and without AI-support.
Timing	The reader study was online from November 17th, 2022, to February 2nd, 2023, without any gaps
Data exclusions	We excluded incomplete tests.
Non-participation	Of 101 readers who started the reader study, 12 did not complete a single test and gave no reasons for non-participation
Randomization	Participants were not allocated into experimental groups. For each experiment, images were randomly selected as described in the Methods section

Reporting for specific materials, systems and methods

We require information from authors about some types of materials, experimental systems and methods used in many studies. Here, indicate whether each material, system or method listed is relevant to your study. If you are not sure if a list item applies to your research, read the appropriate section before selecting a response.

Materials & experimental systems

n/a	Involved in the study
☒	☐ Antibodies
☒	☐ Eukaryotic cell lines
☒	☐ Palaeontology and archaeology
☒	☐ Animals and other organisms
☒	☐ Clinical data
☒	☐ Dual use research of concern

Methods

n/a	Involved in the study
☒	☐ ChIP-seq
☒	☐ Flow cytometry
☒	☐ MRI-based neuroimaging
